# Strong Born—A First of Its Kind National FASD Prevention Campaign in Australia Led by the National Aboriginal Community Controlled Health Organisation (NACCHO) in Collaboration with the Aboriginal Community Controlled Health Organisations (ACCHOs)

**DOI:** 10.3390/ijerph21010085

**Published:** 2024-01-11

**Authors:** Robyn Williams, Sarah Hayton, Annabel Campbell, Holly Kemp, Dorothy Badry

**Affiliations:** 1 Curtin Medical School, Faculty of Health Sciences, Curtin University, Bentley 6102, Australia; 2 National Aboriginal Community Controlled Health Organisation (NACCHO), Canberra 2601, Australia; annabelecampbell@gmail.com (A.C.); holly.kemp@naccho.org.au (H.K.); 3 Faculty of Social Work, University of Calgary, Calgary, AB T2N 1N4, Canada; badry@ucalgary.ca

**Keywords:** health promotion, cultural security, FASD, Aboriginal and Torres Strait Islander, Aboriginal Community Controlled Health Organisations

## Abstract

The Strong Born Campaign (2022–2025) was launched by the National Aboriginal Community Controlled Health Organisation (NACCHO) in 2023. Strong Born is the first of its kind national Aboriginal and Torres Strait Islander health promotion campaign to address Fetal Alcohol Spectrum Disorder (FASD) within Australia. Strong Born was developed to address a longstanding, significant gap in health promotion and sector knowledge on FASD, a lifelong disability that can result from alcohol use during pregnancy. Utilizing a strengths-based and culturally sound approach, NACCHO worked closely with the Aboriginal Community Controlled Health Organisations (ACCHOs) to develop the campaign through co-design, as described in this paper. Since its inception, the ACCHOs have continually invested in driving change towards improvements in Aboriginal health determinants and health promotion. The Strong Born Campaign developed culturally safe health promotion tool kits designed for the community and health sector staff and also offered communities the opportunity to apply for FASD Communications and Engagement Grants to engage in local campaign promotion. The tool kits have been disseminated to 92 ACCHOs across Australia. This paper describes the development of the Strong Born Campaign and activities following its launch in February 2023 from an Indigenous context within Australia, as described by NACCHO.

## 1. Introduction

The focus of Strong Born was on co-creating a culturally informed campaign on FASD for the Aboriginal community in Australia through co-design and community engagement. NACCHO has long been aware of the health and social impacts of FASD within the Aboriginal community, and this was a driving force in developing the Strong Born Campaign launched on 22 February 2023. In the development of the Strong Born Campaign, it is important to note that collaborating with the ACCHO sector was crucial, given their key role in addressing essential health needs and disparities for the Aboriginal community. The ACCHOs continue to lead the way in delivering culturally secure primary health care to Aboriginal and Torres Strait Islander communities in Australia [1]. The ACCHOs contribute to improving Aboriginal and Torres Strait Islander health and wellbeing through the provision of comprehensive primary health care and by integrating and coordinating care and services. ACCHOs are best placed to offer services for Aboriginal people with disabilities as they are already trusted providers of primary health care, given that there remains a gap in culturally safe mainstream providers in Australia [2]. Notably, it is recognized that there is low engagement of Aboriginal people in comparison to non-Aboriginal people in mainstream disability services [2,3,4]. As FASD is a lifelong disabling condition, the ACCHOs are a critical partner in the Strong Born Campaign, given their key role in the delivery of primary health care to Aboriginal people in Australia.

The Strong Born Campaign has been delivered in genuine partnership with ACCHOs and was led by NACCHO. NACCHO is the national peak body representing 145 ACCHOs throughout Australia. NACCHO liaises with its membership and the eight state/territory affiliates, governments, and other organisations on Aboriginal and Torres Strait Islander health and wellbeing, policy and planning issues, and advocacy relating to health service delivery, health information, research, public health, health financing, and health programs. The Strong Born Campaign, led by NACCHO, is grounded in Aboriginal cultural knowledge, strengths, and approaches related to health promotion and harm reduction associated with alcohol use during pregnancy. Additionally, this campaign is strongly aligned with the National Agreement on Closing the Gap [5] policy, specifically Priority Reform 2 on capacity building of the community-controlled sector.

Fetal Alcohol Spectrum Disorder (FASD) is a global health issue and a leading cause of developmental disability worldwide [6]. The prevention of FASD remains a significant global concern, and alcohol use during pregnancy can affect the health of both mother and child [7]. Whilst health promotion approaches in FASD have improved globally, the prevalence of drinking alcohol during pregnancy among the general population has been reported to be 14% in Canada, 30% in the United States, 33% in the United Kingdom, 83% in Russia and 89% in Ireland [8]. It is reported that alcohol consumption during pregnancy ranges from 40–80% in Australia [9].

### 1.1. Strong Born and Public Health Recognition of FASD

FASD is the leading cause of intellectual disability in the world [10]. FASD is a lifelong disability and requires a whole-of-community response. The launch of the Strong Born Campaign signals a shift in the need to respond to FASD as a key public health concern in Australia, recognizing that there are distinct needs within the Aboriginal community that have largely gone unsupported. Effective approaches to FASD need to be tailored to the priorities and preferences of specific populations in Australia, and the need exists to incorporate holistic approaches that recognize the impact of intergenerational trauma [11]. Existing gaps that were reported in current health promotional material on FASD are that materials did not include men and extended family members [12]. It is also important to recognize and situate FASD in the historical landscape of colonization in Australia and to acknowledge its impact on Aboriginal people. The Stolen Generations are those First Nations (Aboriginal and Torres Strait Islander) children who were forcibly removed and separated from their families from the mid-1800s up to the 1970s [13]. The impact of the Stolen Generations continues to ripple throughout the country and across generations today and is a clear factor in alcohol misuse [14].

**Figure 1:**
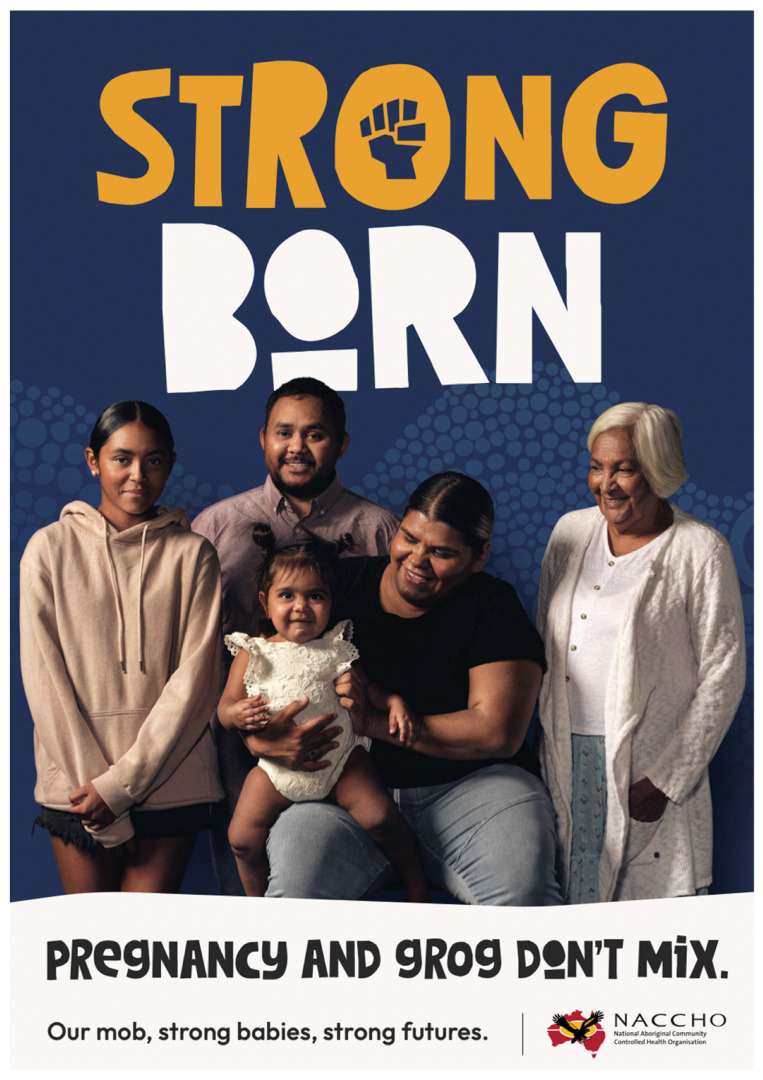
Strong Born Campaign poster.

**Figure 2:**
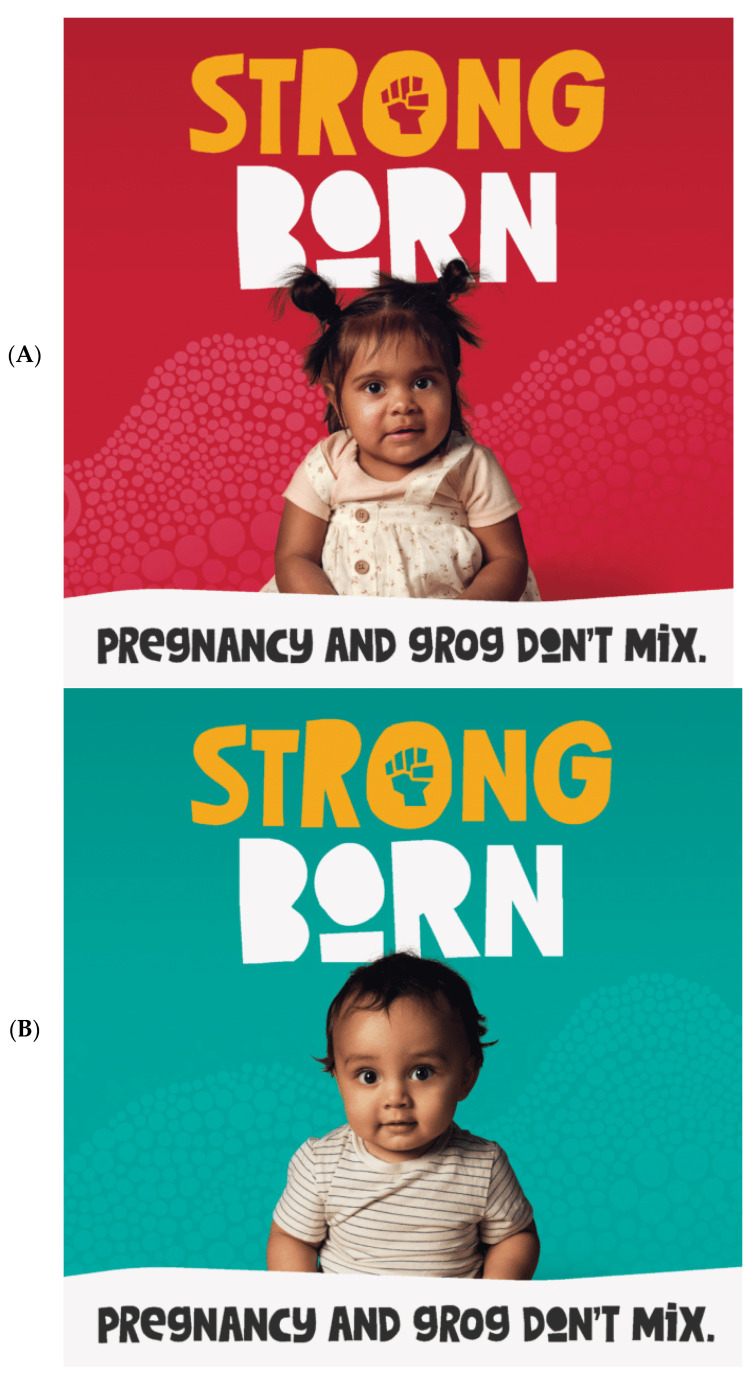

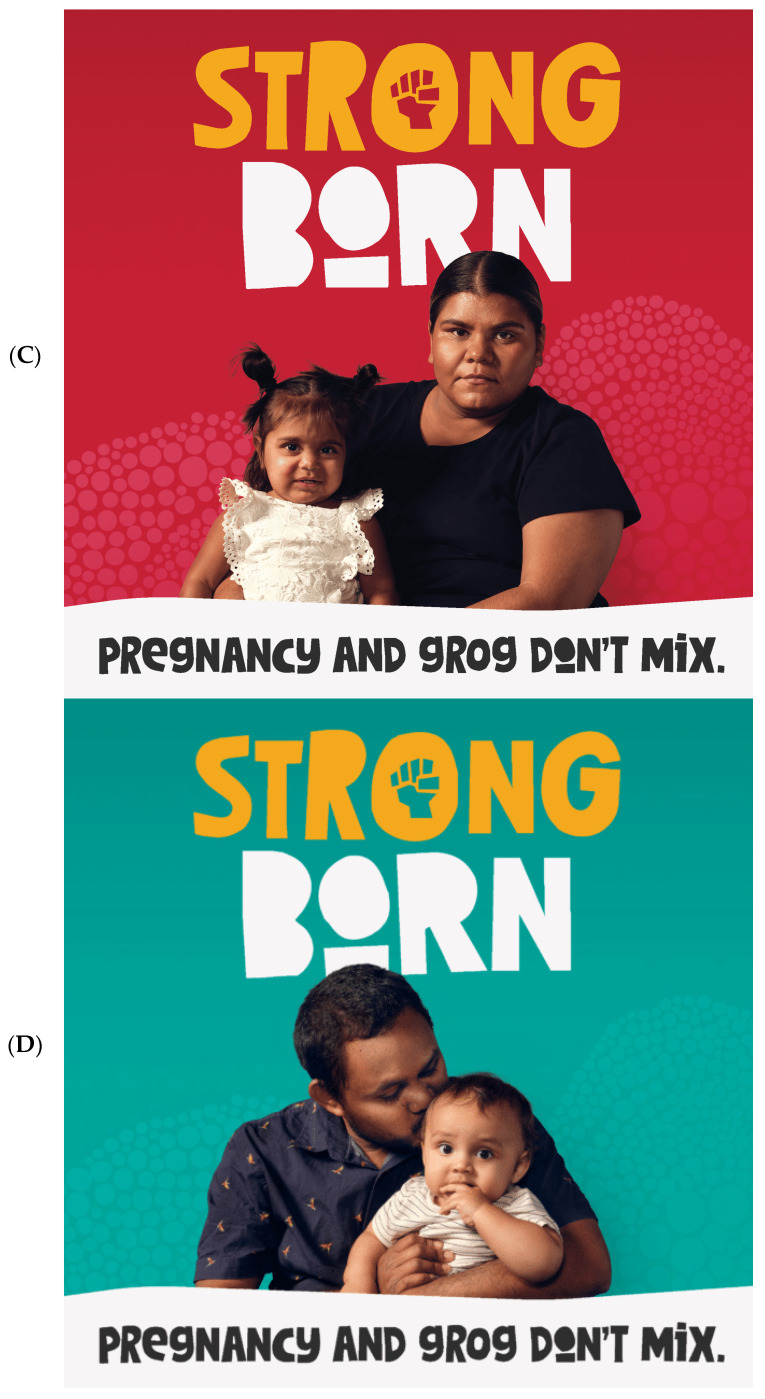
(**A**–**D**) Strong Born Campaign social media tiles.

The Strong Born Campaign is viewed as a critical launching point for creating awareness as well as an impetus to spark yarning about FASD across the country, from the most remote communities to urban settings. The campaign is aligned with the National Information Campaign for Pregnant and Breastfeeding Women and supported by the Foundation for Alcohol Research and Education (FARE) through a partnership launched in 2020, as well as the Commonwealth Department of Health and Aged Care [15]. FARE (2022) reports that FASD is the leading cause of non-genetic disabilities in Australia [15]. Key activities in the Strong Born Campaign include the development and dissemination of toolkits for those in rural, remote, and metro regions of Australia, members and affiliates, and the provision of grants to ACCHOs across Australia. A National FASD Working Group was also established that worked closely with NACCHO throughout the development of the campaign and all materials. Figure 1 below shows one of the Strong Born posters distributed in the campaign, and Figure 2 shows the social media tiles produced for the campaign, and all resources are accessible and downloadable to the community.

### 1.2. The Aims of the Strong Born Campaign Include

▪ Raise awareness of FASD and the harms of drinking alcohol while pregnant among Aboriginal and Torres Strait Islander people in rural and remote communities.
▪ Utilize strength-based messaging with an overall focus on health and strong babies to drive a whole-of-community approach (promoting everyone having a role in growing a strong next generation).
▪ Reduce stigma and shame experienced by women.
▪ Deliver messaging that is safe for people with FASD and their families.

### 1.3. The Development of the Strong Born Campaign Materials

A key component of the Strong Born Campaign was the development of resources in 2022–2023 that were appealing, relevant, and user-friendly for communities. With this in mind, Strong Born developed the following materials for communities and health professionals: Health Professionals Booklet [16], Community Booklet [17], posters [18], and social media tiles [19]. The National FASD Working Group included 17 members with representation from across Australia, and this group played a key role in reviewing and assessing the accuracy of the health promotion messages, and all the materials produced in the campaign. A working document was accessible to all members of the working group, and feedback and editing of the materials was an iterative process that took place over six months, as needed, until a final product was agreed upon.

An example of core messages provided in the Health Professionals Booklet [16] is provided below. Core topics addressed include the following areas: neurological, cognitive, and spiritual, and a brief explanation of each area is provided for the reader. What follows the explanation is a clear message that is provided for the health professional as follows:

Neurological—Alcohol Consumption during pregnancy can cause defects in the development of the central nervous system. This can lead to long term cognitive and behavioural issues… People with FASD need you to be calm and patient, so they can learn and do things at their own speed.

Cognitive—People with FASD need you to be calm, listen and take time with them so they feel heard and understood.

Spiritual—People with FASD might like you to take time to be with them during cultural gatherings and explain what is happening so they can feel more connected.

Another key topic in the Health Professionals Booklet [16] is Yarning with Pregnant Women, which provides core messages on creating a safe space, the importance of taking time in building a trusting relationship and encouraging yarning with trusted others such as Aunties and Elders. A key message about not just focusing on substance abuse is also provided. In many ways, these materials reflect important sound bites for engaging in dialogue and conversations with others and provide a starting place to talk about difficult issues in a respectful way. A core question identified is—“What are you most excited about, being pregnant?” [16] (p. 12). Another example of suggested ways to yarn includes the question, “Would you feel more comfortable if we had one of our health workers in here while we talk, or would like a friend or family member here as well?” [16] (p. 12). The Health Professionals Booklet materials produced by Strong Born are meant to be used at a grassroots level to engage in yarning with people in the community using relational approaches by health care providers. Critical topics include Yarning with Other Community Members, which recognizes the role that partners can have in influencing “if a mum drinks or not” and suggests “yarning through the benefits of them (the partner) not drinking while mum is pregnant” [16] (p. 13).

In a similar fashion, the Community Booklet [17] was developed for use by all members of the community, and a clear message on the front page of the booklet states, “No grog during pregnancy is best.” A core message is Growing Strong Born Babies, stating, “Our bubs need us to love them and to make them feel safe and secure. From the beginning and when bubs are born, remember to stay strong, eat good tukka and connect to Country. Your mob, community and health workers are here to help you while baby is growing and when they’re born” [17] (p. 1). Using common vernacular was critical in this document, and in addressing alcohol use and pregnancy, the question is asked, “What happens when you charge up while pregnant?” Core responses about what is true or not true follow this question with answers such as, “Not all babies will get FASD, but they might if you charge up.” [17] (p. 4). Throughout the Community Booklet, explanations are provided about impacts in areas such as learning, social, emotional, and spiritual, and a clear message is provided, “Don’t be shame to get help” with a graphic of an elder stating, “Let’s have a cuppa and yarn” [17] (p. 9). The materials and messages were clearly developed to support community engagement, to encourage and be welcoming of individuals seeking support and help in an inviting way. An important message provided on the last page of the booklet focuses on who can support you and your family during pregnancy. The responses state:

“There are so many people who will help to keep you and bub strong and healthy while pregnant. Grog and pregnancy don’t mix but if you find it hard to stop drinking you can yarn with your clinic mob. Having a bub and dealing with life can be hard. Be kind to yourself and your loved ones. Work together to make small changes first for a healthy bub and family. Clinic mob want to help and yarn through what you need.”

[17] (p. 12)

Other important resources developed included posters [18] and social media tiles [19]. Each poster stated, “Pregnancy and grog don’t mix” and each poster had one of the following four core messages:

▪ Let’s make every baby strong born.
▪ Our mob, strong babies, strong futures.
▪ No alcohol during pregnancy is best.
▪ Safe pregnancies are men’s business too.

A core message underlying the Strong Born Campaign was that everyone in the community has a role to play in responding to and preventing FASD. A YouTube promotion video (1 min 30 s) on the Strong Born Campaign to raise awareness on FASD provides a brief visual overview of the development of the campaign and materials and is available online—https://youtu.be/Jwfy1w35Zvg (accessed on 20 November 2023). The campaign also offers a long-form video for health professionals, providing a focus on the importance of yarning about alcohol use and pregnancy in a safe and non-judgmental way, recognizing that FASD is also men’s business—https://www.youtube.com/watch?v=eia-UQRfa9E (accessed on 20 November 2023). This video also identifies the diagnostic pathway and the potential for NDIS (National Disability Insurance Scheme) support and suggests that all community members have a role in supporting babies to be Strong Born. Other activities undertaken in supporting engagement with the campaign materials included implementation and info webinars, where participants were invited in the months of July, August, and September of 2023 to join in order to learn about the toolkits and to share ideas about what is working in different communities.

The Strong Born Campaign offered a robust approach to engagement with the materials by providing a campaign launch in February 2023, offering webinars to explain the campaign, providing toolkits to communities, providing YouTube videos on Strong Born, and offering community grants to localize campaigns in various communities across Australia.

### 1.4. Australian Context of Health Promotions on FASD

FASD continues to be poorly understood and underserviced in mainstream health services in Australia [20]. Health professions play an important role in the prevention of alcohol-exposed pregnancies, and alcohol use has been reported as common among pregnant women in Australia [21]. An Australian study in 2013 reported that 1 in 4 Australian women continued to drink alcohol during pregnancy, and only 53% abstained during the entire pregnancy. In Western Australia, it was reported that 23% of Aboriginal women consumed alcohol during pregnancy [12]. When seeking information on alcohol use and pregnancy, women prefer to ask health professionals, and yet, it is noted that health professionals in Australia have been hesitant to discuss alcohol consumption due to the lack of adequate health promotional resources to provide to clients or patients [12]. Another Australian study found that the majority of children born with FASD were found to have a sibling with FASD, indicating missed opportunities for prevention within the same family [21]. It was stated that FASD has become the most unrecognized and misunderstood disability in Australia [22]. This has far-reaching consequences for children and adults who are living with what is often undiagnosed FASD.

### 1.5. Australian Indigenous Context and FASD

Prior to colonization, Aboriginal people had no word in their language to identify disability [3,23]. A key concern identified in the literature is that the lack of strength-based approaches in responding to FASD has likely contributed to the ongoing stigma experienced by individuals living with this disability [24]. In Australia, new narratives are emerging that counter mainstream deficit narratives and recognize the inherent strengths-based foundation of Aboriginal-led responses to both disability and FASD [25]. Responding to FASD from a strengths-based perspective has been highlighted in the leadership and advocacy of Aboriginal communities in Australia in response to FASD, as reflected in the uptake of Strong Born across the country. The Strong Born Campaign represents significant agency and advocacy by the Aboriginal community and provides not only the Aboriginal community but the wider community with a framework to mitigate stigma that often plays a pivotal role in the lack of diagnosis and services for children and adults with FASD [26]. The Strong Born Campaign offers a distinct and unique opportunity to engage in new yarning about FASD in Australia and to frame critical responses that are grounded in Aboriginal voices and communities across the country. As authors, we acknowledge that FASD is not an Indigenous problem; however, the intersection of colonization, disability, and systematic racism continues to influence devastating and predictable pathways for those with FASD, including the over-representation of Indigenous children and youth in child protection and justice settings [27,28].

### 1.6. Aboriginal and Torres Strait Islander Leadership

NACCHO recognizes and acknowledges the longstanding leadership of many Aboriginal people and communities responding to FASD over the past few decades in Australia, including the pioneering advocacy of Dr. Jan Hammill [29], Dr. Lorian Hayes [30], and the Aboriginal community from Fitzroy Crossing community [31]. In this context, Aboriginal communities have a history of mobilizing and seeing past the stigma of FASD and recognizing the strengths in the child and the individual [25]. This response of seeing beyond the challenges associated with FASD also reflects the strengths of Aboriginal culture and the different worldview on disability [28]. This work has considered Aboriginal approaches to health as well as an Indigenous worldview of disability.

### 1.7. Social and Emotional Wellbeing of Aboriginal and Torres Strait Islander People with Disability and Their Families

Acknowledging the importance of good social and emotional wellbeing (SEWB) of Aboriginal people in Australia is also critical [32], as culture is increasingly recognized as an important part of interventions [33]. Under the lens of good SEWB, it is essential to recognize and support children with FASD who have often experienced early adversity, including being raised in child protection care. Without effective support and interventions, children and adults with FASD remain vulnerable and at risk of early onset of mental health conditions and higher rates of suicidal ideation [34]. Often, individuals and families receive limited support, placing an increased burden on families, and it has been reported that more informal caring occurs within Aboriginal families in comparison to non-Aboriginal families [32]. It is important for families caring for children or relatives with FASD to be supported as much as possible, and it is hoped that increased awareness of FASD will support better outcomes for children and families.

### 1.8. Campaign Implementation: Framing Strong Born from an Indigenous Lens

In brief, the scope of the Strong Born campaign is broader than a traditional prevention lens as it supports engagement in prevention and supporting children and adults with FASD. This is a major departure from mainstream health promotion campaigns on FASD, where the focus is generally on the prevention of FASD through alcohol reduction during pregnancy. In effect, the Strong Born Campaign applies a novel and holistic approach with two key facets, including (1) prevention of FASD and (2) identifying the importance of supporting children and adults with FASD in the community through creating increased awareness of FASD. The main means of supporting communities to respond to FASD is the offering of community-based grants from NACCHO to promote local applications of the Strong Born Campaign, including the need to provide information on services and supports available for children and adults with FASD. This is particularly important, as most children and adults with FASD remain undiagnosed in Australia [22]. Whilst prevention of FASD remains critically important, the need also exists to apply a more holistic framework that will enhance advocacy and support communities in the best way forward for improving quality of life through access to services for children and adults with FASD. The provision of community-based grants was seen as one way to mobilize local support for children and families by providing information on what support is available, as well as the development of local resources and responses to FASD.

## 2. Materials and Methods

### Approaches to the Strong Born Campaign

The development of Strong Born involved a multi-stage process that included utilizing co-design as a key theoretical framework [35] informing the approach to the campaign. Co-design, as applied in Strong Born, recognizes the development of a genuine partnership and collaboration between NACCHO, ACCHOs, and the established National Aboriginal and Torres Strait Islander FASD Working Group (the FASD working group). To ensure that NACCHO understood the FASD campaign landscape, a comprehensive material analysis report was undertaken internally to examine previous FASD campaigns. This process included an audit of previous FASD campaigns targeted at Aboriginal communities in rural and remote areas. The results of the audit included the identification of persistent gaps in health promotions on FASD in the sector and deficit-based approaches. They underscored the urgent need to develop materials that could be used by health professionals on FASD that were culturally safe and appropriate. It was recognized that FASD prevention campaigns often only focus on women’s behavior around alcohol consumption during pregnancy and provide messages such as alcohol and pregnancy do not mix but often do not take a whole-of-community approach [36]. The focus of the Strong Born Campaign, informed by the audit, was to be as inclusive of the community as possible.

The audit was followed by consultations led by NACCHO with a range of sector staff within ACCHOs that included Alcohol and other Drugs (AOD), disability, SEWB, child and maternal health, and FASD researchers and cultural experts from across Australia. This consultation process identified what was working effectively in the Aboriginal community in FASD health promotion. A key theme that emerged was the urgent and ongoing need for training of all ACCHO staff on FASD. Critically, the consultation process identified the importance of:

▪ Utilizing face-to-face engagements between multi-disciplinary ACCHO staff and community members as a key campaign delivery channel.
▪ Increasing the confidence of ACCHO staff to discuss FASD with community members by providing detailed culturally-specific health professionals information so staff could deliver the health messaging in face-to-face engagements.
▪ Recognizing the role of partners, friends, family, and other community members in preventing FASD and supporting a healthy pregnancy reflects the preference for whole-of-community approaches that do not stigmatize women.
▪ Making Strong Born Campaign materials publicly available and easily accessible. Social media tiles of all the poster portraits and messages were provided online for anyone to access, download, and share or post on social media or online, making materials completely accessible to the community.

Other important activities undertaken in the development and review of the Strong Born Campaign materials included focus group testing pre-launch and the review of the materials at an Aboriginal Medical Service post-launch.

A focus group was undertaken in Western Australia in August 2022, with 6 Aboriginal participants to review all campaign materials for the soundness of core messages and to identify gaps or corrections required. Dr. Robyn Williams conducted this focus group with six individuals, all Aboriginal women in Western Australia, to review and test out these core messages. The focus group consisted of two young adult women, two Aunties, one Elder, and a health professional. All women played many roles in their families and communities, and this group provided critical feedback on finalizing the four core messages for the posters.

The Strong Born Campaign materials were shared in a presentation to Derbarl Yerrigan Health Service (DYHS) on FASD post-launch on 14 April 2023 in Perth, Western Australia, and a rich discussion took place with a diverse group of participants. Those attending included Aboriginal and non-Aboriginal health staff, including general practitioners, Aboriginal health practitioners, social workers, and nurses. The workshop was also attended by young Aboriginal women (young mothers and single women) in the community. The participants identified that they found the materials to be culturally safe in the messaging on alcohol and drug use during pregnancy in an urban Aboriginal community. Young women attending the workshop also commented on the importance of understanding the impacts of alcohol during pregnancy and the impact of FASD on children without support and intervention in place, particularly as they commence school.

As the Strong Born Campaign is grounded in providing support to the community, as noted above, it was essential to utilize a co-design process in the project design. The FASD Working Group was established by NACCHO and included multi-disciplinary staff from ACCHOs, ACCOs, cultural and clinical FASD experts, and researchers, including representation from remote, rural, regional, and metropolitan areas nationally. The membership of the FASD Working Group reflected the diversity amongst Aboriginal and Torres Strait Islander cultures in Australia and was chaired by NACCHO’s Deputy CEO, Dr. Dawn Casey. The working group met with NACCHO every two months during the project development and provided guidance on the materials to be utilized in the Strong Born campaign. After the first working group meeting, it was identified that there was a need to support ACCHOs in developing strength and place-based solutions within the campaign design that were sensitive to potential stigma against pregnant women. NACCHO worked with the Foundation for Alcohol Research and Education (FARE) to restructure the program funding for only a small portion of funds to go toward developing national campaign materials, with the majority of funding allocated to two grant rounds to enable ACCHOs to deliver localized solutions.

National campaign materials included branding and associated tag lines, posters, social media tiles, a health professionals’ booklet, and a community booklet. Strong Born toolkits were built for rural and remote ACCHOs that included printed copies of the booklets, posters, workforce polo shirts, and a USB with all social media and poster templates. The NACCHO team also provided support for ACCHOs to develop place-based materials and messaging by developing ‘How to Guides’ for customization of the templates and conducting a series of online webinars. An example of the Strong Born Campaign poster (Figure 1) is included below.

In April 2023, NACCHO launched one of two grant rounds to support ACCHOs to deliver place-based solutions. Key communication for the campaign was NACCHO’s social media channels and direct correspondence with ACCHOs by the NACCHO Executive.

## 3. Results

### 3.1. Findings: Impact of the Strong Born Campaign

The Strong Born Campaign was launched in February of 2023, and the descriptive findings reported here offer some insight into the impact of the Strong Born nationally. These findings reflect internal, descriptive research related to activities undertaken within NACCHO after Strong Born was launched. The following activities have taken place on the Strong Born Campaign:

▪ Strong Born was launched on 22 February 2023 online via Microsoft Teams by NACCHO CEO Pat Turner and Caterina Giorgi, accompanied by the FASD Working Group members Emily Carter, Sue Thomas, and Dr. Robyn Williams, who gave context to Aboriginal leadership in the prevention of FASD and support of people with FASD and shared perspectives from the ACCO sector.
▪ The campaign resources were launched on the same day and received overwhelmingly positive feedback, with a highly engaged audience of 200 people from across Australia, New Zealand, Canada, and the USA attending the launch. Strong Born was featured in seven publications (news articles, radio, interviews, and news channels).
▪ Launch of the Strong Born Campaign on the NACCHO website, inclusive of a series of fact sheets, online resources, and downloadable copies of all campaign collateral. Campaign materials are all available on the NACCHO website: https://www.naccho.org.au/fasd/strong-born/ (accessed on 20 November 2023).
▪ Since its launch, 5047 people have visited the Strong Born website hosting campaign resources, and 92 toolkits have been sent out to communities across the country in rural, remote, and urban communities.

### 3.2. Anecdotal Feedback on the Strong Born Campaign to NACCHO Received as Follows

▪ We have been sharing material on social media since the campaign launch. We’ve been seeing very good engagement; the brand and style are very strong. Congratulations—campaign webinar attendee.
▪ A very important resource, LinkedIn comment.
▪ Strong Born is a great campaign with some great resources. I look forward to spreading the word, campaign webinar attendee.
▪ Thanks for all the amazing work that has gone into producing such great, appropriate and culturally secure material. I look forward to working with it.—ACCHO worker.
▪ Strong Born is a great campaign and much needed—campaign webinar attendee.

### 3.3. Feedback from Strong Born Webinar Launched in Australia on 22 February 2023

▪ Loving the final product. Really proud to have been part of the working group. It was such a great group to be part of, and NACCHO led out on this fantastically. Communication and feedback were on point. I am keen to get campaign material out to community.
▪ Fantastic launch for a very important issue, congratulations to all involved, really looking forward to using your awesome campaign resources, congratulations to all.
▪ The resources look fantastic, great achievement and congratulations to your team.
▪ Congratulations to the NACCHO team and the National FASD Working Group. It is wonderful to see the resources in today’s office launch, we can’t wait to share and promote these.
▪ Thank you for sharing, I have been working in the awareness and understanding of FASD space in NZ for over 11 years and have had the pleasure of meeting a couple of you over this time. It has been a long journey with good outcomes and attribute this to engaging our te ao Maori Tikanga, traditional practices. Loved hearing the journey of your Kaupapa I feel inspired in what I do.

### 3.4. Community Engagement with Strong Born through Campaign Toolkits and FASD Grants

Since the launch on 22 February 2023, Strong Born campaign toolkits have been disseminated to 92 rural and remote ACCHOs across Australia and affiliate representatives across all jurisdictions. The toolkits delivered directly to communities consist of the Health Professionals Booklet [16], the Community Booklet [17], posters [18], and social media tiles [19]. Communities were also invited to apply for FASD Communications and Engagement Grants to NACCHO. The key goals of the grants are (1) to raise awareness of the harms of alcohol consumption during pregnancy, (2) to support people living with FASD and their families by developing knowledge and understanding of FASD and services that can help, and (3) to support communities in the development of their own culturally appropriate materials that are relevant in their region or community. The overall intent is to support localized responses and the development of place-based communication materials within local communities designed to respond to local needs.

Grant round 1 resulted in 24 ACCHOs across very remote, remote, and rural areas across the nation receiving funding, which resulted in place-based campaign materials being developed in communities. Grant recipients were encouraged to make the campaign their own and reflective of the needs in their community. The success of the Strong Born launch and immediate engagement with the campaign resulted in NACCHO receiving additional funding to increase grant opportunities, expand the program to metro communities, and deliver animated video versions of the information booklets. Examples of the ways in which grants were utilized include the following:

▪ Campaign materials being translated into local Aboriginal languages.
▪ Development of several local community champions campaigns, further enhancing the whole-of-community approach.
▪ Awareness and community day activities

Examples of the Strong Born Campaign posters [18] and social media tiles [19] are provided below. Figure 1 is the Strong Born Campaign poster. Figure 2 shows the social media tiles produced for the campaign. In addition to the campaign posters and social media tiles, a Health Professional Booklet [16] and Community Booklet [17] are available as described above. All resources are readily available online for anyone to download at www.naccho.org.au/fasd/strong-born/ (accessed on 20 November 2023).

## 4. Discussion

The Strong Born Campaign was launched on 22 February 2023 after a well-planned and highly committed process of community engagement with the ACCHOs led by NACCHO. It was recognized that FASD is a significant public health issue impacting Aboriginal communities across Australia that is deeply linked to the history of colonization [27,28]. As a recognized public health issue, FASD has not received adequate resourcing, support, and funding as a disability, which is critical in relation to prevention and intervention. Children and families living with FASD continue to be overrepresented in child protection and justice settings, and the opportunity to lead and increase Aboriginal agency in responding to FASD is impacted by a lack of resourcing [31]. Whilst Aboriginal leadership in responding to FASD in Australia has been established, Strong Born reflects an authentic investment into responding to FASD by bringing the dialogue to the people through this campaign. It is believed and has already been demonstrated that Strong Born will open dialogue and provide resources that will positively contribute to improving quality of life, improved SEWB, and improved opportunities in the prevention of FASD in Australia. In raising awareness of FASD as a key public health issue, as has been done through the Strong Born Campaign, it also follows that it is critical to recognize that community-based support is essential to support children and families effectively. This further highlights the need for effective responses and increased resources in access to screening and diagnosis for FASD.

Prior to the Strong Born Campaign, there was a major gap in the provision of culturally safe and appropriate health promotional materials on FASD for the Aboriginal community in Australia. This is further compounded by the fact that FASD training for relevant social service sectors is generally not mandatory [37], underscoring the urgency and need for a national campaign to create awareness and provide resources to communities. Under the lens of decolonizing disability and ongoing capacity building, it is also important that culturally safe FASD training is delivered to local communities by Aboriginal people whenever possible. Whilst training was outside of the scope of the Strong Born Campaign, the campaign encourages agencies to incorporate FASD training for local agencies. FASD remains a complex and sensitive topic that is largely stigmatized both in Australia and globally [38,39]. Poor sector awareness of FASD, combined with stigma, plays a significant role in the lack of early access to diagnosis and intervention for children and adults with FASD. Strong Born holds the possibility of changing the narrative on the Australian response to FASD through grassroots community engagement and offers new and innovative opportunities through awareness and knowledge to improve the lives of children and adults living with this disability.

Co-design was utilized in Strong Born as its guiding principles ensure the engagement of all stakeholders in planning, design, and decision-making with a focus on desired outcomes. Co-design is a flexible approach that takes into consideration social and cultural determinants of health and recognizes the power differentials resulting from colonization [40]. In the past, FASD prevention work has largely focused on professionals as they are responsible for the delivery of the information. In the Strong Born Campaign, and as a result of using a co-design approach, the information on prevention has been geared towards the community as well as towards professionals. Campaign booklets were designed with both community members and professionals in mind, providing relevant information to both sectors. Co-design was the method chosen for this campaign, and it was informed by a comprehensive process involving the establishment and engagement of a national FASD working group. Guiding principles governed the work conducted. There were multiple levels of cooperative governance between the NACCHO, the ACCHOs, and the National FASD Working Group established for Strong Born, leading up to the campaign development, delivery, and expansion.

What is also new in the Strong Born Campaign is the use of local vernacular within the Aboriginal community in Australia. The term for alcohol is different around the country; it could be grog, it could be another term using the local language, and there are still many Indigenous languages and dialects that utilize their own terminology. The downloadable Strong Born social media templates can be customized by communities to suit their needs. Communities can apply for grants to actualize the campaign locally, which supports its sustainability. This approach provides the opportunity for a much deeper engagement in the Strong Born Campaign, as communities can take local ownership of how the campaign unfolds.

The work of Strong Born is not just talking about prevention. The brochures also focus on supporting children and adults with FASD, which is unique to the Strong Born Campaign and a product of the co-design process. It is critical that this information be provided to the community on how to support individuals living with FASD. It is also critical to recognize the undiagnosed population of individuals living with FASD who have never had the benefit or opportunity to be assessed and diagnosed [22].

The original premise of the Strong Born Campaign was to focus on rural and remote communities. However, based on feedback from the FASD working group and the fact that limited infrastructure exists to respond to FASD in Australia, it was clear that the campaign had to expand and include metro areas. Notably, FASD is not solely an Indigenous problem or located in a single geographical area. FASD remains a serious concern in all communities where there is limited awareness about the impact of alcohol consumption during pregnancy. Future research includes NACCHO undertaking a comprehensive evaluation of the Strong Born Campaign.

## 5. Conclusions

The national Strong Born Campaign on FASD is the first of its kind campaign that applies a holistic approach to FASD in the Aboriginal and Torres Strait Islander communities in Australia. Strong Born offers a cultural framework to support responding to the stigma of FASD. In summary, NACCHO has delivered 92 Strong Born community toolkits across Australia, and 24 ACCHOS have received community grants. Due to the success and uptake of the campaign, NACCHO was successful in obtaining additional funding to increase community grants and expand the campaign to metro areas in Australia. Additional funding supported the development and creation of an animated version of the campaign booklets available on YouTube. Strong Born is an important tool in raising awareness and supporting people living with FASD, their families, and carers across Australia.

Importantly, the Strong Born campaign has the potential to enhance the capacity of the ACCHO sector further and to provide the catalyst for early access to diagnosis, intervention, and quality of life for children and adults living with FASD in Australia.

## Data Availability

All data collected is contained within the article.

## Abbreviations

ACCO	Aboriginal Community Controlled Organisations
ACCHOs	Aboriginal Community Controlled Health Organisations
FASD	Fetal Alcohol Spectrum Disorder
FARE	Foundation for Alcohol Research and Education
NACCHO	National Aboriginal Community Controlled Health Organisation
SEWB	Social and emotional wellbeing
